# Quality of life assessment tools for chronic cough in children: a systematic review

**DOI:** 10.3389/fpubh.2025.1515858

**Published:** 2025-05-21

**Authors:** Bingxue Cao, Tianhan Wang, Siming Zhai, Sha Zhang, Bin Yuan

**Affiliations:** ^1^The Affiliated Hospital of Nanjing University of Chinese Medicine, Nanjing, Jiangsu, China; ^2^Department of Pediatrics, Kunshan Hospital of Traditional Chinese Medicine, Suzhou, Jiangsu, China; ^3^Department of Pediatrics, Yixing Traditional Chinese Medicine Hospital Affiliated to Nanjing University of Chinese Medicine, Wuxi, Jiangsu, China; ^4^First Clinical Medical College, Nanjing University of Chinese Medicine, Nanjing, Jiangsu, China

**Keywords:** chronic cough, children, life quality, cultural modification, systematic review

## Abstract

**Purpose:**

The aim is to examine studies evaluating the life quality of children suffering from chronic cough over the last 10 years, concentrating on three areas: the development and validation of new tools, the cultural adaptation of existing tools, and the usage of common tools.

**Methods:**

A series of digital explorations were conducted in PubMed, Web of Science, EMBASE, and the Cochrane Library databases to find pertinent literature. The selection of literature adhered to specific criteria for its inclusion and exclusion. Analytical methods are documented in the International Prospective Register of Systematic Reviews, identified by the registration number CRD42024583481.

**Results:**

Our collection encompassed 3,186 records, culminating in the inclusion of 14 studies. One study concentrated on the development and validation of a new assessment tool. The study evaluated the dependability, accuracy, and responsiveness of the child cough specific quality of life questionnaire (CC-QoL), but the number of participants was notably small. Research focusing on the creation and verification of localized language editions of current tools was absent. Thirteen studies, predominantly from China and Australia, employed quality of life (QoL) evaluation instruments as the outcome metrics. Chinese studies employed a wide range of evaluation instruments. Research in Australia employed the parent cough specific quality of life questionnaire (PC-QoL).

**Conclusion:**

Over the last 10 years, minimal research has been conducted on the creation, application, and cultural modification of QoL evaluation instruments for chronic cough in children.

**Systematic review registration:**

https://www.crd.york.ac.uk/PROSPERO/view/CRD42024583481.

## 1 Introduction

Coughing commonly manifests as a symptom, frequently necessitating medical advice for respiratory diseases. Research shows that over 75% of children seek medical advice more than five times a year due to coughing, with 14% of these consultations surpassing 15 (1). Actions like these can drastically reduce the well-being of children and their parents, leading to pressures on mental, financial, and medical resources (2).

Currently, tools used to evaluate chronic cough in children fall into two classifications: objective and subjective. Objective evaluation tools primarily focus on measuring cough sensitivity and tracking how often coughs occur. The initial approach lacks standardized methods for assessing cough sensitivity, while the latter relies mainly on automated and semi-automated devices, susceptible to factors such as microphone type and placement, recording conditions, study participants, and non-cough-related sounds. Additionally, observing coughs can alter how a person perceives coughing, imposes specific limitations, and has not been integrated into conventional clinical practices yet (3). As a result, current assessments of coughs are largely based on subjective evaluation tools.

Following a literature review prior to December 2013, the Chest Cough Expert Panel recommends using quality of life (QoL) assessment tools as the benchmark for age-related measurements (4). For adolescents aged 14 and above, employing reliable and recognized QoL assessment surveys such as the Leicester cough questionnaire (LCQ) (5) and the cough specific quality of life questionnaire (CQLQ) (6) is recommended as primary tools for measuring cough impacts. The recommendation is to use the parent cough specific quality of life questionnaire (PC-QoL) (7, 8) for children younger than 14 years (4). Customized measurement tools or questionnaires for evaluating coughs can assess the unique impact and intensity on the QoL of a child or caregiver, playing a vital role in determining the severity of the condition and the effectiveness of clinical interventions (4).

Ten years have elapsed since the Chest Cough Expert Panel last conducted a thorough analysis. This study aims to scrutinize and evaluate research over the past 10 years. The focus is given to three principal domains: the development and validation of novel tools; the cultural adaptation of these tools (local language versions of existing tools); and the use of conventional tools to evaluate the QoL of children with chronic cough. This systematic review was based on the Preferred Reporting Items for Systematic Reviews and Meta-Analyses (PRISMA) statement, reported in 2020 (9). The procedures for analysis are recorded in the International Prospective Register of Systematic Reviews, marked by the registration number CRD42024583481. The analysis of the connection between QoL assessment tools and health outcomes is beyond the scope of this review; thus, no meta-analysis has been conducted.

## 2 Methods

### 2.1 Retrieval strategy

We retrieved data from PubMed, Web of Science, EMBASE, and The Cochrane Library. The basic search strategy was (Adolescent OR Adolescence OR Teen OR Teenage OR Youth OR Child) AND (Leicester Cough Questionnaire OR LCQ OR Quality of Life Questionnaire OR QLQ OR Cough Impact OR CCIQ OR Visual Analog Scales OR VAS) AND (Chronic Cough OR Cough Variant Asthma OR CVA OR Postinfectious Cough OR PIC OR Upper Airway Cough Syndrome OR UACS OR Post-Nasal Drip Syndrome OR PNDS OR Protracted Bacterial Bronchitis) s OR PBB OR protracted infectious bronchitis OR PIB OR Atopic cough OR AC OR Eosinophilic bronchitis OR EB OR Gastroesophageal reflux-related cough OR GERC, retrieval time range: January 2014 to December 2023 (past 10 years); language range: unrestricted.

### 2.2 Inclusion and exclusion criteria

#### 2.2.1 Inclusion criteria

(1) Study groups: <19 years of age; (2) Study populations with chronic cough symptoms (cough duration > 4 weeks); (3) Interviewees: children/adolescents self-reporting, parent/caregiver reporting, or both.

#### 2.2.2 Exclusion criteria

(1) Lack of basic data; (2) Animal experiments, cell experiments, case reports, meta-analyses, summaries, reviews, incomplete or unfinished studies; (3) Typical asthma, cystic fibrosis, and tuberculosis.

### 2.3 Review process

#### 2.3.1 Filtration of the documentation

Two reviewers (Reviewer A: Bingxue Cao, Reviewer B: Siming Zhai) methodically assessed the remaining documents using EndNote, focusing on relevance. The initial evaluation considered the reading topic, summary, and keywords, discarding any documents unrelated to the research. The final selection hinged on the rest of the documentation, which was determined through a thorough search and full-text review.

#### 2.3.2 Documentation quality evaluation

Each of the two reviewers conducted a quality review of the final included documents, followed by verification, consultation, and ultimately, the assessment of the literature’s quality. We used the Cochrane Risk of Bias version 2 (RoB2, reviewed in 2019) (10) for randomized controlled trials (RCTs) and the Methodological Index for Non-Randomized Studies (MINORS) (11) for non-RCTs.

#### 2.3.3 Information extraction

We standardized a table and extracted the following information from the literature: Literature, tools, country or region, age (years), number of samples, interviewees, and disease name. Studies on cultural adaptation or the creation and validation of new tools gathered additional data, including dimensions and numbers, reliability, validity, sensitivity, and the minimally important difference (MID).

During the entire review phase, any differences or inquiries between reviewers regarding the literature need to be resolved through negotiation and concluded. All pending matters should be deliberated with the third reviewer (Reviewer C: Tianhan Wang).

## 3 Results

### 3.1 Characteristics included in the study

**Figure 1:**
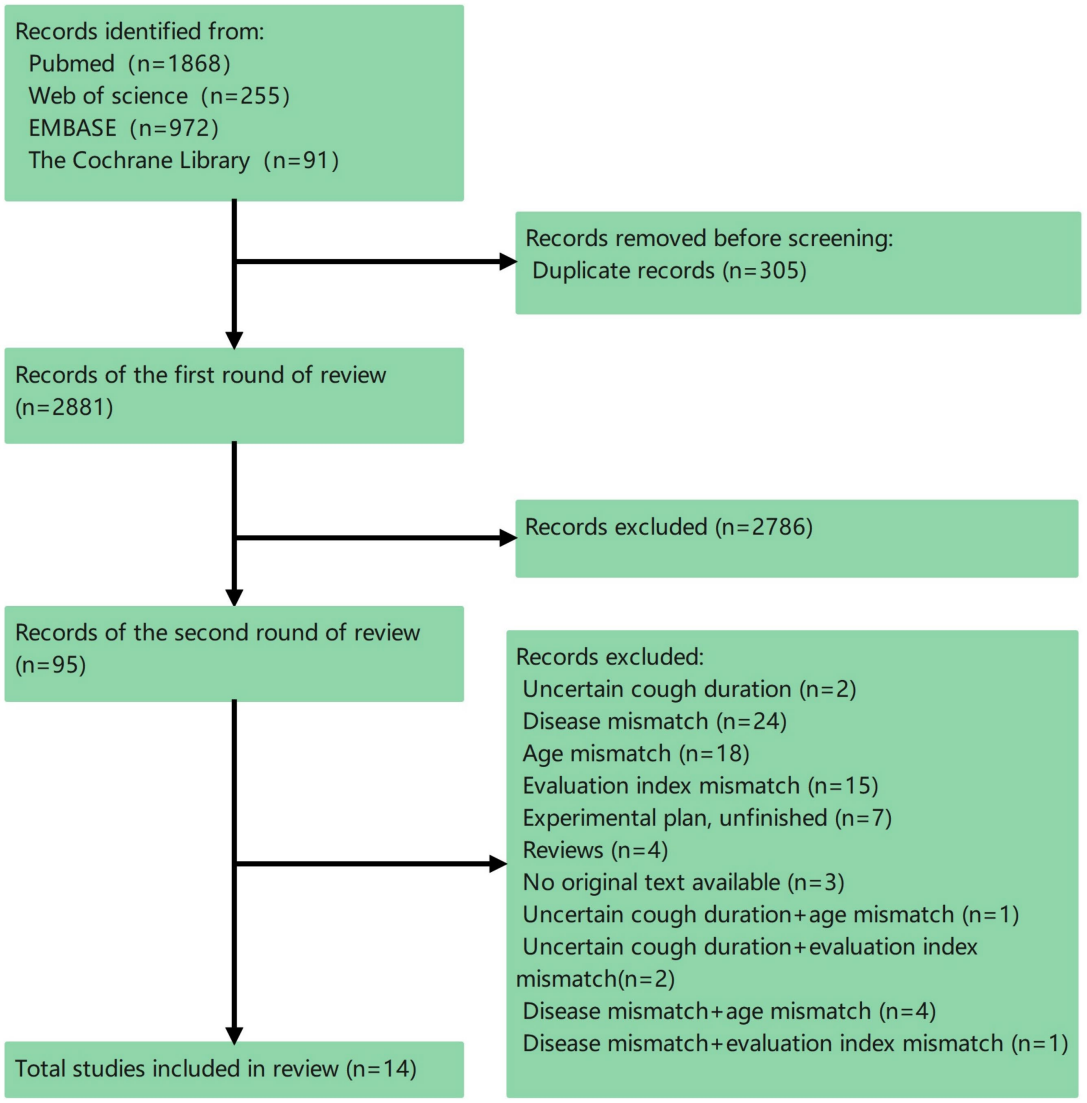
System evaluation process diagram.

The total count of records in our collection reached 3,186, leading to the selection of 14 different studies (Figure 1). Of the 14 studies incorporated, 1 involved the creation and validation of a novel evaluation tool; 13 were clinical trials employing the QoL assessment tool as the ultimate measure. No cultural adaptation studies of existing tools have been found.

**Figure 2:**
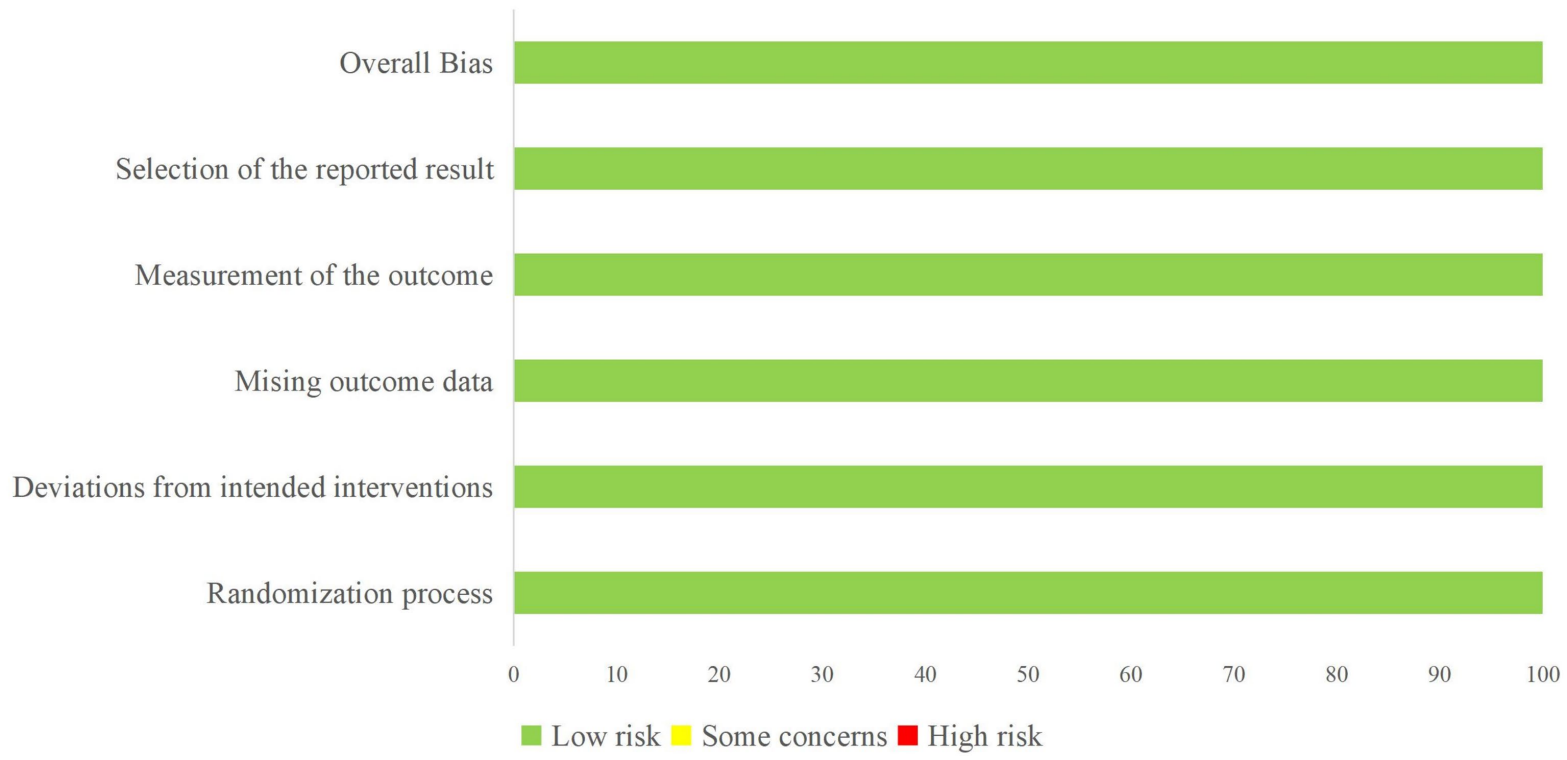
Outcomes of quality evaluations in RCT studies (horizontal axis %).

All included studies underwent a risk of bias assessment: 3 were RCTs (12–14) (Figure 2), while 11 were non-RCTs. The latter included a total of 4 controlled (15–18) (Table 1) and 7 non-controlled studies (19–25) (Table 2).

**Table 1:** Outcomes of quality evaluations in non-RCTs (comparative studies).

Literature	Item 1	Item 2	Item 3	Item 4	Item 5	Item 6	Item 7	Item 8	Item 9	Item 10	Item 11	Item 12	Total score
Zhou X 2016 (15)	2	2	0	1	0	2	0	0	0	2	2	2	13
PA Newcombe 2016 (16)	2	0	1	2	0	0	2	0	2	2	2	2	15
Wang ZH 2020 (17)	2	0	0	2	0	2	2	0	2	2	2	2	16
WANG L 2021 (18)	2	0	1	2	0	2	2	0	2	2	2	2	17

Item 1: A clearly stated aim. Item 2: Inclusion of consecutive patients. Item 3: Prospective collection of data. Item 4: Endpoints appropriate to the aim of the study. Item 5: Unbiased assessment of the study endpoint. Item 6: Follow-up period appropriate to the aim of the study. Item 7: Loss to follow up less than 5%. Item 8: Prospective calculation of the study size. Item 9: An adequate control group. Item 10: Contemporary groups. Item 11: Baseline equivalence of groups. Item 12: Adequate statistical analyses.

The items were scored 0 (not reported), 1 (reported but inadequate), or 2 (reported and adequate).

**Table 2:** Outcomes of quality evaluations in non-RCTs (non-comparative studies).

Literature	Item 1	Item 2	Item 3	Item 4	Item 5	Item 6	Item 7	Item 8	Total score
Gao F 2019 (19)	2	0	2	2	0	2	2	0	10
Yu X 2019 (20)	2	0	1	2	0	0	2	0	7
Loyie-Toon, Y. G. 2019 (21)	2	2	1	2	0	2	2	0	11
McCallum GB 2020 (22)	2	2	1	2	1	2	0	0	10
Prime, S. J. 2021 (23)	2	0	1	2	0	2	2	0	9
Rosen, R. 2017 (24)	2	1	1	2	0	0	2	0	8
Marchant JM 2021 (25)	2	0	1	2	0	0	2	0	7

Item 1: A clearly stated aim. Item 2: Inclusion of consecutive patients. Item 3: Prospective collection of data. Item 4: Endpoints appropriate to the aim of the study. Item 5: Unbiased assessment of the study endpoint. Item 6: Follow-up period appropriate to the aim of the study. Item 7: Loss to follow up less than 5%. Item 8: Prospective calculation of the study size.

The items were scored 0 (not reported), 1 (reported but inadequate), or 2 (reported and adequate).

### 3.2 Information excerpt

#### 3.2.1 Research on the development and validation of new tools

Comprising 16 items, the Child Cough specific Quality of Life Questionnaire (CC-QoL) specifically assesses the QoL for children with cough (Table 3). Every item is evaluated on a 7-level Likert scale, with the assessments reflecting the previous week. Elevated scores signify improved QoL. Nonetheless, the confirmation relied on a comparatively limited number of samples (16).

**Table 3:** Information collection form for the development and validation study of the new tool.

Literature	Tools	Country or region	Age (years)	Number of samples	Interviewees	Disease name	Dimensions and numbers	Reliability	Validity	Sensitivity	MID
PA Newcombe 2016 (16)	CC-QoL	Australia	10 (8, 12)	130	Self-report by children/adolescents	Chronic cough	Physical (7 items), psychological (6 items), and social (3 items)	Cronbach’s alpha = 0.94, ICC = 0.39 ~ 0.45	The overall construct validity and criterion validity of the scale were evaluated by analyzing its relationship with four additional scales: VCD, VAS, PedsQL, and SCAS. Findings showed a notable link between the full scale and VCD, VAS, and PedsQL, excluding the social aspect of PedsQL post-intervention. Regarding SCAS, only the symptoms of panic and social phobia that were reported showed a significant correlation with CC-QoL.	The full scale, along with scores in each domain, is displayed, showing a significant improvement in children’s QoL reported after intervention (*p* < 0.001).	It ranged from 0.37 ~ 1.36 (distribution-based approach) to 1.11 ~ 1.58 (anchor-based approach). A MID of 1.1 is recommended.

Age (years): As outlined in literature (16), the median (interquartile range) [M (P25, P75)] is used.

CC-QoL, child cough specific quality of life questionnaire; ICC, intraclass correlation coefficient; VCD, verbal category descriptive scale; VAS, visual analog scale; PedsQL, pediatric quality of life inventory; SCAS, Spence Children’s Anxiety Scale.

#### 3.2.2 Research employing quality of life evaluation as a metric for results

The primary studies included in the research were mainly from China and Australia (Table 4). Chinese literature has the most research on cough variant asthma. In Australia, three studies focus on bronchiectasis, while the other two studies are about non-specific chronic cough. While Australian studies uniformly utilized PC-QoL, Chinese studies employed a variety of assessment tools. The majority of research conducted in China failed to identify the interviewees. Within these studies, one employed a comprehensive evaluation questionnaire for children’s QoL, and another combined a psychological questionnaire with PC-QoL. No research existed in which evaluations were exclusively conducted by children/adolescents.

**Table 4:** Information collection form for clinical studies using QoL assessment tools as outcome measures.

Literature	Tools	Country or region	Age (years)	Number of samples	Interviewees	Disease name
Boonjindasup, W. 2023 (12)	STAI, PC-QoL	Australia	11.3 ± 3.5/10.6 ± 3.9	54/52	②	Chronic cough
Wan, J. 2021 (13)	LCQ, CSS	China	10.04 ± 1.17/9.95 ± 1.13	64/64	Unclear	CVA
Cao JY 2023 (14)	CSS	China	7.86 ± 1.56/8.36 ± 1.28	65/65	Unclear	CVA
Wang ZH 2020 (17)	VAS, CSS	China	7.70 ± 2.29/8.15 ± 2.03	60/52	Unclear	CVA
WANG L 2021 (18)	LCQ, CSS	China	8.05 ± 1.30/8.11 ± 1.22	100/100	② and doctors	CVA
Zhou X 2016 (15)	*Mean score of cough	China	9.2 ± 1.1/9.2 ± 1.2	368/529	Unclear	CVA
Gao F 2019 (19)	VAS	China	8.9 ± 0.8	103	① or ②	UACS
Yu X 2019 (20)	VCD	China	9.3 ± 1.1	118	① and ②	Chronic cough
Loyie-Toon, Y. G. 2019 (21)	PC-QoL, CC-QoL	Australia, New Zealand	8.7(5.4, 11.3)	85	① (for children >7 years of age) and ②	BT
McCallum GB 2020 (22)	PC-QoL	Australia, Alaska, New Zealand	12.3 ± 2.6	131	②	CSLD、BT
Prime, S. J. 2021 (23)	PC-QoL	Australia	1.85(1.23, 3.81)	91	②	Chronic wet cough
Rosen, R. 2017 (24)	PedsQL	America	6.5 ± 3.7	77	②	GERD
Marchant JM 2021 (25)	PC-QoL-8	Australia	5.8(3.5, 8.4)	152	②	BT

STAI, state–trait anxiety inventory; PC-QoL, parent cough specific quality of life questionnaire; LCQ, leicester cough questionnaire; CSS, cough symptom score; VAS, visual analog scale; VCD, verbal category descriptive scale; CC-QoL, child cough specific quality of life questionnaire; PedsQL, pediatric quality of life inventory; CVA, cough variant asthma; UACS, upper airway cough syndrome; BT, bronchiectasis; CSLD, chronic suppurative lung disease; GERD, gastroesophageal reflux disease.

Interviewees: ① = self-report by children/adolescents; ② = proxy report by parents/guardians.

Age (years): As outlined in scholarly works (12–15, 17–20, 22, 24), the mean ± standard deviation (–x ± s) is used; whereas in literature (21, 23, 25), the median (interquartile range) [M (P25, P75)] is used.

The control experiment details age and sample size in this manner: the experimental group provides the initial half of the data, while the control group contributes the latter half, interspersed with a “/” space.

*Mean score of cough: mean score of daytime cough = (sum of daytime frequency scores + sum of daytime cough severity scores)/total number of days; mean score of nighttime cough = (sum of nighttime frequency scores + sum of sleep disorder scores due to nighttime cough)/total number of days. Total symptom score = mean score of daytime cough + mean score of nighttime cough. There was a lack of corroborative literature for this rating, which might have been developed experimentally by researchers.

## 4 Discussion

### 4.1 Tools for evaluating the quality of life in children with chronic cough

#### 4.1.1 Development and validation of new tools

A high-quality questionnaire should meet high standards in both reliability and validity (26–28). Within the realm of questionnaire analysis, reliability serves as a measure of the genuineness of the attributes measured, categorized into internal and external reliability (28). The first indicates how consistent the questionnaire items are internally, whereas the second shows how consistent the questionnaire is in repeated measurements (28). Methods for assessing reliability include Cronbach’s alpha coefficient and test–retest reliability (29, 30). The validity is indicative of the precision of the measurement outcomes and the veracity of the content reflected. Enhancing validity involves a more precise recording of the respondents’ thoughts and behavioral characteristics. PA Newcombe’s research identified a Cronbach’s alpha coefficient exceeding 0.8 as the threshold for high reliability. The aggregate Cronbach’s alpha coefficient proved to be adequate, and the test–retest reliability evaluation received a moderate rating (details not included). Concurrently, the CC-QoL shows an effective correlation with other QoL assessment tools and cough detection techniques, demonstrating a high sensitivity to variations (16).

For illnesses mainly marked by coughing, the CC-QoL may act as an essential tool to evaluate its effects, yet the questionnaire’s effectiveness still requires additional verification. The assessment outcomes of this system also reveal a limited practical application of the questionnaire.

#### 4.1.2 Cultural adaptation of existing tools

The Chest Cough Expert Panel recommends keeping the QoL assessment questionnaire for chronic cough unchanged. If alterations are necessary, it is essential to reassess the accuracy and reliability of the revised version. One should not assume uniform performance of QoL assessment questionnaires across various studies, cultures, and populations. The conversion of current questionnaires into a different language necessitates techniques such as forward and backward translation, cognitive interviews, etc., to preserve the validity of the content and other measurement aspects, as well as the disclosure of procedures and findings (4). Studies in this field are comparatively scarce in China. *Clinical practice guidelines for the diagnosis and management of children with cough in China (version 2021)* suggest that the domestic pediatric field should prioritize the development and validation of cough-specific QoL scales suitable for Chinese children (31). Regrettably, the systematic review did not incorporate any studies that focused on the development and validation of localized language versions of existing tools.

#### 4.1.3 Usage of tools

Studies in China evaluating the QoL for children suffering from chronic cough often face challenges such as variable scale quality, unclear applicability to certain groups, and inconsistent scale usage. Chinese studies demonstrated heterogeneity in the selection of assessment tools, incorporating instruments such as the LCQ, visual analog scale (VAS), verbal category descriptive scale (VCD), and cough symptom score (CSS). It should be noted that while the VAS shows potential for quantifying cough severity, its application in this field remains problematic due to limited evidence supporting measurement accuracy and inconsistent correlations with other validated cough metrics (4). Among the 7 studies reviewed, 5 exhibited ambiguity in specifying respondents (4 omitted this critical methodological detail, and one ambiguously permitted both). These factors could affect the accuracy of the evaluation results. There is a lack of studies in which assessments are exclusively conducted by children/adolescents, an aspect also affected by the characteristics of existing evaluation tools.

### 4.2 Tool selection

#### 4.2.1 Evaluation content

The PedsQL program is suitable for children and adolescents aged 2 to 18 (32, 33). This questionnaire is used to assess the general aspects of QoL, facilitating the comparison of QoL between sick children and healthy ones (33, 34). This method is extensively employed in disease research due to the lack of precise QoL measurement instruments. Nonetheless, generic QoL assessment instruments might fail to effectively detect symptom variations in specific illnesses. Moreover, they are generally inappropriate for evaluating the adverse reactions of therapies pertaining to particular illnesses. Conversely, assessment instruments tailored to specific diseases demonstrate superior explanatory capacity for patients on a personal level. Investigators ought to choose suitable scales in line with clinical trial goals, considering all-encompassing factors, and when required, integrate two evaluation instruments.

#### 4.2.2 Interviewees

The QoL is a multifaceted concept that requires assessing every aspect of life and personal experiences, encompassing the effects of sickness and therapy (35). In the case of young children, reporting by parents/guardians is often considered a discerning method of management (36). However, the assessment results reflect parents/guardians’ perceptions regarding the impact on the child’s QoL. There is ample evidence suggesting that the information provided by proxy respondents is not equivalent to the information reported by the patients themselves (37). Children aged over 7 years might have the capability to describe their QoL (4). Suitable evaluation instruments should be chosen according to the child’s age profile. The CC-QoL, derived from children’s self-reports, has been formulated, yet additional verification is still necessary. In the case of teenagers aged 14 or older, established and dependable QoL questionnaires serve as viable substitutes.

## 5 Conclusion

Research on the development, application, and cultural adaptation of QoL evaluation instruments for children suffering from chronic cough is limited in this systematic review. The outcome leads us to reassess our assessment methodology and prompts a reassessment of the goals in clinical studies. The main focus of clinical research is on exploring the influence of various treatment approaches on clinical elements, frequently overlooking QoL as a minor issue. The integration of QoL evaluation in every experiment lacks dependability; yet, when QoL assessment is considered suitable, integrating pertinent outcome metrics ought to be a crucial phase in the design of clinical trials, not merely a secondary consideration. For maintaining the scientific integrity and impartiality of QoL evaluations, a thorough examination of the research goals, the demographic studied, and the nature of the assessments is essential. It’s vital to meticulously choose and integrate QoL evaluation instruments relevant to this research, ensuring their uniform application. It is only at this juncture that we can genuinely and thoroughly grasp the QoL conditions, thereby offering more dependable data backing for clinical application.

It should be acknowledged that this study has some limitations. The literature search was limited to a relatively small number of databases and did not include Chinese databases. Additionally, due to the research timeline, the latest studies might not have been included. These factors may introduce bias in the evaluation. Future research should expand database coverage and establish a more comprehensive evaluation system.

## Data Availability

The original contributions presented in the study are included in the article/supplementary material, further inquiries can be directed to the corresponding author.

## References

[1] Marchant JM Newcombe PA Juniper EF Sheffield JK Stathis SL Chang AB. What is the burden of chronic cough for families? Chest. (2008) 134:303–9. doi: 10.1378/chest.07-2236, PMID: 18641100

[2] Bergmann M Haasenritter J Beidatsch D Schwarm S Hörner K Bösner S . Coughing children in family practice and primary care: a systematic review of prevalence, aetiology and prognosis. BMC Pediatr. (2021) 21:260. doi: 10.1186/s12887-021-02739-4, PMID: 34088294 PMC8176681

[3] Hall JI Lozano M Estrada-Petrocelli L Birring S Turner R. The present and future of cough counting tools. J Thorac Dis. (2020) 12:5207–23. doi: 10.21037/jtd-2020-icc-003, PMID: 33145097 PMC7578475

[4] Boulet LP Coeytaux RR Mccrory DC French CT Chang AB Birring SS . Tools for assessing outcomes in studies of chronic cough: CHEST guideline and expert panel report. Chest. (2015) 147:804–14. doi: 10.1378/chest.14-2506, PMID: 25522203 PMC5991766

[5] Birring SS Prudon B Carr AJ Singh SJ Morgan MD Pavord ID. Development of a symptom specific health status measure for patients with chronic cough: Leicester cough questionnaire (LCQ). Thorax. (2003) 58:339–43. doi: 10.1136/thorax.58.4.339, PMID: 12668799 PMC1746649

[6] French CT Irwin RS Fletcher KE Adams TM. Evaluation of a cough-specific quality-of-life questionnaire. Chest. (2002) 121:1123–31. doi: 10.1378/chest.121.4.1123, PMID: 11948042

[7] Newcombe PA Sheffield JK Juniper EF Petsky HL Willis C Chang AB. Validation of a parent-proxy quality of life questionnaire for paediatric chronic cough (PC-QOL). Thorax. (2010) 65:819–23. doi: 10.1136/thx.2009.133868, PMID: 20805179

[8] Newcombe PA Sheffield JK Chang AB. Minimally important change in a parent-proxy quality-of-life questionnaire for pediatric chronic cough. Chest. (2011) 139:576–80. doi: 10.1378/chest.10-1476, PMID: 20947650

[9] Page MJ McKenzie JE Bossuyt PM Boutron I Hoffmann TC Mulrow CD . The PRISMA 2020 statement: an updated guideline for reporting systematic reviews. BMJ. (2021) 372:n71. doi: 10.1136/bmj.n71, PMID: 33782057 PMC8005924

[10] Liu J Liu C Hua C. Risk bias assessment tool RoB2 (revised version 2019) for randomized controlled trial: an interpretation. Chin J Evid Based Med. (2021) 21:737–44. doi: 10.7507/1672-2531.202011144

[11] Slim K Nini E Forestier D Kwiatkowski F Panis Y Chipponi J. Methodological index for non-randomized studies (minors): development and validation of a new instrument. ANZ J Surg. (2003) 73:712–6. doi: 10.1046/j.1445-2197.2003.02748.x, PMID: 12956787

[12] Boonjindasup W Marchant JM McElrea MS Yerkovich ST Masters IB Chang AB. Does routine spirometry impact on clinical decisions and patient-related outcome measures of children seen in respiratory clinics: an open-label randomised controlled trial. BMJ Open Respir Res. (2023) 10:e001402. doi: 10.1136/bmjresp-2022-001402, PMID: 37169400 PMC10186453

[13] Jun W Zhou Y Meng-tian S Xiang-zheng Y. Clinical efficacy on Erchentang combined with Sanzi Yangqintang in treatment of cough variant asthma in children with phlegm evil accumulation lung syndrome. Chin J Exp Tradit Med Formul. (2021) 27:58–63. doi: 10.13422/j.cnki.syfjx.20210624

[14] Cao JY Wang YC Deng XX. Efficacy of β2-adrenergic receptor agonist combined with corticosteroid in the treatment of children with cough variant asthma. World J Clin Cases. (2023) 11:7610–8. doi: 10.12998/wjcc.v11.i31.7610, PMID: 38078144 PMC10698450

[15] Zhou X Hong J Cheng H Xie J Yang J Chen Q . Budesonide suspension nebulization treatment in Chinese pediatric patients with cough variant asthma: a multi-center observational study. J Asthma. (2016) 53:532–7. doi: 10.3109/02770903.2015.1111903 26517446

[16] Newcombe PA Sheffield JK Petsky HL Marchant JM Willis C Chang AB. A child chronic cough-specific quality of life measure: development and validation. Thorax. (2016) 71:695–700. doi: 10.1136/thoraxjnl-2015-207473, PMID: 26842959

[17] Wang Z Peng H Rao K. The secondary prevention effect and influence on serum sIgG4, IL-27 and IL-33 levels of subcutaneous immunotherapy in children with allergic rhinitis and cough variant asthma. J Clinical Otorhinolaryngol, Head, Neck Surg. (2020) 34:793–8. doi: 10.13201/j.issn.2096-7993.2020.09.007, PMID: 33040502 PMC10127719

[18] Liang W Na H Hongwei L Yuejing S Shuying B. Effect of Hanchuan Zupa granules combined with montelukast sodium in treatment of cough variant asthma in children and its effect on airway inflammation, immunoglobulin, and leukotriene levels. Drug Eval Res. (2021) 44:1016–21.

[19] Gao F Gu QL Jiang ZD. Upper airway cough syndrome in 103 children. Chin Med J. (2019) 132:653–8. doi: 10.1097/cm9.0000000000000118, PMID: 30855345 PMC6416099

[20] Yu X Kong L Jiang W Dai Y Wang Y Huang L . Etiologies associated with chronic cough and its clinical characteristics in school-age children. J Thorac Dis. (2019) 11:3093–102. doi: 10.21037/jtd.2019.07.36, PMID: 31463138 PMC6687982

[21] Lovie-Toon YG Grimwood K Byrnes CA Goyal V Busch G Masters IB . Health-resource use and quality of life in children with bronchiectasis: a multi-center pilot cohort study. BMC Health Serv Res. (2019) 19:561. doi: 10.1186/s12913-019-4414-5, PMID: 31409413 PMC6693266

[22] McCallum GB Singleton RJ Redding GJ Grimwood K Byrnes CA Valery PC . A decade on: follow-up findings of indigenous children with bronchiectasis. Pediatr Pulm. (2020) 55:975–85. doi: 10.1002/ppul.24696, PMID: 32096916

[23] Prime SJ Carter HE McPhail SM Petsky HL Chang AB Graves N . Chronic wet cough in Australian children: societal costs and quality of life. Pediatr Pulm. (2021) 56:2707–16. doi: 10.1002/ppul.25438 33939893

[24] Rosen R Mitchell PD Amirault J Amin M Watters K Rahbar R. The edematous and erythematous airway does not denote pathologic gastroesophageal reflux. J Pediatr. (2017) 183:127–31. doi: 10.1016/j.jpeds.2016.11.035, PMID: 27979581 PMC7885125

[25] Marchant JM Cook AL Roberts J Yerkovich ST Goyal V Arnold D . Burden of Care for Children with bronchiectasis from parents/Carers perspective. J Clin Med. (2021) 10:5856. doi: 10.3390/jcm10245856, PMID: 34945152 PMC8707334

[26] Bannigan K Watson R. Reliability and validity in a nutshell. J Clin Nurs. (2009) 18:3237–43. doi: 10.1111/j.1365-2702.2009.02939.x, PMID: 19930083

[27] Gélinas C Loiselle CG Lemay S Ranger M Bouchard E McCormack D. Theoretical, psychometric, and pragmatic issues in pain measurement. Pain Manag Nurs. (2008) 9:120–30. doi: 10.1016/j.pmn.2007.12.001, PMID: 18706383

[28] Andrade C. Internal, external, and ecological validity in research design, conduct, and evaluation. Indian J Psychol Med. (2018) 40:498–9. doi: 10.4103/IJPSYM.IJPSYM_334_18 30275631 PMC6149308

[29] Cho E Kim S. Cronbach’s coefficient alpha: well known but poorly understood. Org Res Methods. (2015) 18:207–30. doi: 10.1177/1094428114555994

[30] Green SB. A coefficient alpha for test-retest data. Psychol Methods. (2003) 8:88–101. doi: 10.1037/1082-989X.8.1.88, PMID: 12741675

[31] The Subspecialty Group of Pharmacology tSoP, Chinese Medical Association, Disorders NCRCfCHa, the Subspecialty Group of Respiratory Diseases tSoP, Chinese Medical Association, the Children’s Respiratory Professional Committee tSoPoCMDA, the Editorial Board CJoP. Clinical practice guidelines for the diagnosis and management of children with cough in China (version 2021). Chin J Pediatr. (2021) 59:720–9. doi: 10.3760/cma.j.cn112140-20210513-00423 34645211

[32] Varni JW Sherman SA Burwinkle TM Dickinson PE Dixon P. The PedsQL family impact module: preliminary reliability and validity. Health Qual Life Out. (2004) 2:55. doi: 10.1186/1477-7525-2-55, PMID: 15450120 PMC521692

[33] Varni JW Seid M Kurtin PS. PedsQL™ 4.0: reliability and validity of the pediatric quality of life inventory™ version 4.0 generic core scales in healthy and patient populations. Med Care. (2001) 39:800–12. doi: 10.1097/00005650-200108000-00006, PMID: 11468499

[34] Varni JW Burwinkle TM Seid M. The PedsQL as a pediatric patient-reported outcome: reliability and validity of the PedsQL measurement model in 25,000 children. Expert Rev Pharm Out. (2005) 5:705–19. doi: 10.1586/14737167.5.6.705, PMID: 19807613

[35] Calman KC. Quality of life in cancer patients--an hypothesis. J Med Ethics. (1984) 10:124–7. doi: 10.1136/jme.10.3.124, PMID: 6334159 PMC1374977

[36] Solans M Pane S Estrada MD Serra-Sutton V Berra S Herdman M . Health-related quality of life measurement in children and adolescents: a systematic review of generic and disease-specific instruments. Value Health. (2008) 11:742–64. doi: 10.1111/j.1524-4733.2007.00293.x, PMID: 18179668

[37] Chang PC Yeh CH. Agreement between child self-report and parent proxy-report to evaluate quality of life in children with cancer. Psychooncology. (2005) 14:125–34. doi: 10.1002/pon.828, PMID: 15386781

